# Shared decision-making allows subordinates to lead when dominants monopolize resources

**DOI:** 10.1126/sciadv.aba5881

**Published:** 2020-11-25

**Authors:** Danai Papageorgiou, Damien R. Farine

**Affiliations:** 1 Max Planck Institute of Animal Behavior, Department of Collective Behavior, Universitätsstraße 10, Konstanz 78457, Germany.; 2 University of Konstanz, Department of Biology, Universitätsstraße 10, Konstanz 78457, Germany.; 3 University of Konstanz, Center for the Advanced Study of Collective Behaviour, Universitätsstraße 10, Konstanz 78457, Germany.; 4 Kenya Wildlife Service, P.O. Box 40241-001000, Nairobi, Kenya.; 5 Department of Ornithology, National Museums of Kenya, P.O. Box 40658-001000, Nairobi, Kenya.

## Abstract

The concepts of leadership and dominance are often conflated, with individuals high in the social hierarchy assumed to be decision-makers. Dominants can exclusively benefit from monopolizing food resources and, therefore, induce an intragroup conflict when leading their group to these resources. We demonstrate that shared decision-making reduces such conflicts by studying movement initiations of wild vulturine guineafowl, a species that forms large, stable social groups with a steep dominance hierarchy. When dominant individuals displace subordinates from monopolizable food patches, the excluded subordinates subsequently initiate collective movement. The dominants then abandon the patch to follow the direction of subordinates, contrasting with nonmonopolizable resources where no individuals are excluded, and dominant individuals contribute extensively to group decisions. Our results demonstrate the role of shared decision-making in maintaining the balance of influence within animal societies.

## Teaser

Subordinates lead when dominants monopolize.

## INTRODUCTION

Across the animal kingdom, the power held by dominant individuals mirrors their ability to monopolize resources (*1*, *2*), but not necessarily their influence on group decisions (*3*). When animals exploit food resources collectively, dominant group members often displace subordinates, forcing them to the periphery of the group (*4*, *5*). In theory, this social stratification generates a conflict (*6*): Clumped food resources that can be monopolized are most beneficial for a dominant and least beneficial for the subordinate members of its group, if the latter get excluded. Shared decision-making is predicted to allow groups with stable membership to reduce this conflict (*3*), since all individuals can contribute to determining which resources their group encounters. However, despite being inherently linked, the processes of competitive exclusion and collective decision-making have been largely considered in isolation. For example, the exclusion of subordinates from food resources can generate differences in the state of need among group members, in turn shifting their priorities from group cohesion to moving toward a new resource (*7*, *8*). Understanding the link between social interactions and collective decision-making, driven by resource competition, can illuminate how the balance of power is maintained or lost in animal societies.

## RESULTS

**Fig. 1:**
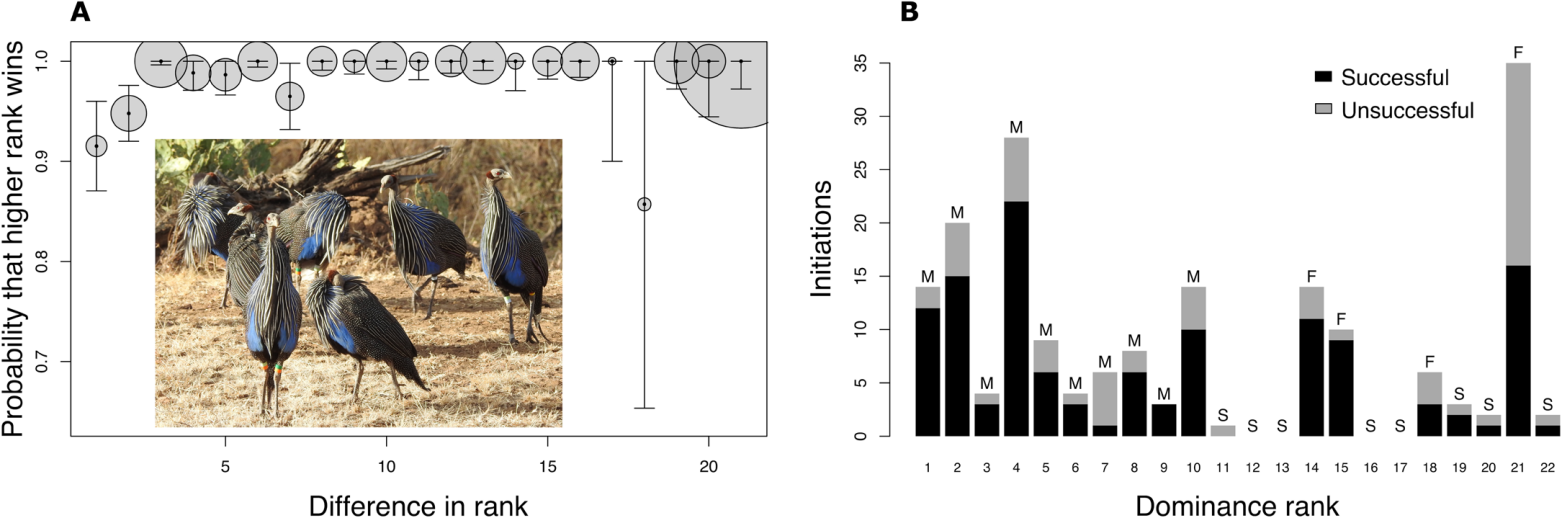
Vulturine guineafowl groups have a steep dominance hierarchy with dominant individuals having more influence over group decisions. (**A**) Higher-ranked individuals have a much higher probability of winning interactions (table S3), even when the difference in rank is small [whiskers: 95% confidence intervals calculated following (*39*); size of circles: number of observations (from 4 to 134) divided by the number of dyads with this difference in rank; see fig. S2 for other groups]. (**B**) Higher-ranked group members are typically more likely to be successful (black bars showing success and gray bars showing failure) at initiating group movements from nonmonopolizable food resources (M: male, F: female, S: subadult; individual 21 was the oldest female in the group). Photo credit: Danai Papageorgiou.

To investigate the relationship between dominance interactions and leadership, we tracked groups of wild vulturine guineafowl (*Acryllium vulturinum*) at the Mpala Research Center, in a Kenyan savannah-woodland ecosystem. This largely terrestrial bird species lives in cohesive, stable, and nonterritorial groups (fig. S1) (*9*). Observations of 2113 dominance interactions across three habituated groups reveal a steep social hierarchy, with males always outranking females (Fig. 1A and fig. S2). Further, observations of 183 group departures (*10*) from natural resources, which cannot be monopolized by one or a few dominant individuals (hereby nonmonopolizable; table S1), suggest that higher-ranked individuals are more successful in initiating group movements [Generalized linear model (GLM): χ^2^ = 8.22, *P* < 0.001, table S2]. However, all adults can initiate group movements successfully (Fig. 1B and fig. S3), complementing growing evidence that, in animal societies, group decisions are, to a large degree, shared (*3*, *11*).

**Fig. 2:**
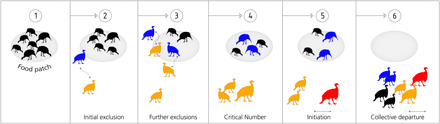
Subordinates initiate group departures when they are excluded from a monopolizable food resource. Initially, (1) all group members enter a patch. (2) Dominant individuals (blue) displace subordinates (orange) who occasionally try to reenter the patch (3). (4) When the number of excluded group members reaches a critical threshold, one excluded bird (red) initiates departure (5). (6) Within a few minutes, all group members follow the direction led by the initiator (red). The direction moved by the initiators then determines subsequent group movements (fig. S4).

The distribution of food resources is often associated with rates of agonistic interactions, with dominant individuals excluding subordinates when resources are clumped (*12*). What are the consequences of such interactions on subsequent group departures? Vulturine guineafowl mainly feed on dispersed seeds and grasses (*13*), but they also feed on monopolizable resources such as insect-rich elephant dung and the fleshy fruit of prickly pear (*Opuntia* spp.). We created experimental monopolizable resources (herein patches), allowing us to record social interactions and subsequent leadership by multiple groups (see table S1). We found that after groups enter a patch, dominant individuals soon displace subordinates, with a number of subordinates accumulating at the periphery. Once reaching a critical number, these excluded subordinates then initiate collective movement away from the patch and are later followed by dominant individuals (Fig. 2).

All individuals enter a patch when a group first arrives. However, subordinates are soon displaced from the resource, with the mean time to first displacement (means ± SD: 4.72 ± 1.79 min after arrival; Fig. 2 and fig. S5A) taking place approximately half the time before the last group member leaves the patch (means ± SD: 8.16 ± 2.85 min). On average, excluded individuals spend more than a minute (means ± SD: 1.28 ± 1.19 min) at the periphery of the patch before departing (Fig. 2), suggesting that initiations and subsequent departures are not an immediate consequence of having lost an agonistic interaction. Not all displacements result in exclusion from the patch, with individuals that are displaced in the beginning of a session being less likely to be excluded to the periphery than those displaced later on (GLM: χ^2^ = 11.51, *P* < 0.001; fig. S5B). Attempts to rejoin the patch after exclusion (fig. S5C) further highlight the idea that individuals do not leave voluntarily.

Across all the observation sessions we conducted at patches (see table S1), we found that successful initiations take place once a critical number of excluded individuals accumulate at the periphery of the patch (means ± SD: 12.71 ± 5.37 individuals; Fig. 2). This critical number, on average 13 (± 5) individuals at the periphery, is independent of group size [Linear Model (LM): *P* > 0.05]. On average, the departure process (from the first departure to the final departure) takes approximately 25% of the time the group spent on the patch (means ± SD: 2.23 ± 1.37 min).

**Fig. 3:**
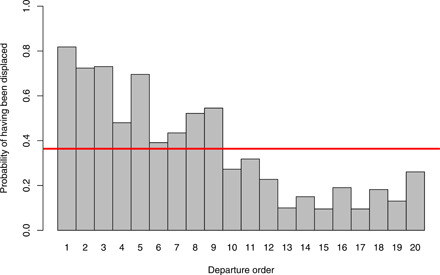
The first individuals to depart from the periphery of monopolizable food resources are more likely to have been aggressed and thus displaced. By contrast, individuals that follow in later positions along the order of departures are less likely to have been displaced. The red line represents the mean probability of having been displaced across all departure orders (data from two habituated groups, see also fig. S6 and table S5).

Data from departures by habituated groups (table S1) reveal no consistency in the identity of the first initiator. However, across all departures from the habituated groups, the three highest-ranked males never once initiated movement (0 of 33 departures, binomial *P* < 0.001; table S4). Instead, we found that, across all departures from all study groups (table S1), the first initiator is almost always an individual that has been previously displaced (excluded individuals initiated in 35 of 41 departures, binomial *P* < 0.001). Further, individuals occupying an early position in the departure order are also disproportionately more likely to have been excluded from the patch, while individuals that occupy later positions in the departure order are less likely to have been excluded (Fig. 3, fig. S6, and table S5).

**Fig. 4:**
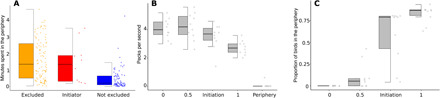
Consequences of being excluded to the periphery of a monopolizable food resource. Each dot represents one individual. (**A**) Excluded individuals (including those that eventually initiated departures) spend substantially more time at the periphery of the patch than individuals that are never excluded (table S6). (**B**) Birds at the periphery do not have access to food and thus peck the ground much less often compared to when they first access the patch (0) and compared to birds that remain on the patch halfway through the group’s presence (0.5), at the moment of the initiation (Initiation), and 10 s before the last bird departs the patch (1; table S7). (**C**) Initiations correspond to a marked increase in the proportion of individuals at the periphery. The variation in the proportion of birds at the periphery at the moment of the initiation is not due to differences in group size, as the number of individuals is more consistent across group sizes (mean = 13 ± 5). Boxes in panels (A-C) represent the lower quartiles, medians, and upper quartiles, with the whiskers showing the range from the lowest values (to a minimum of 1.5 times the range from the first to the third quartile) and the largest values (to a maximum of 1.5 times the range from the first to the third quartile).

Why would the exclusion of group members prompt them to initiate collective movement? Our data suggest that being excluded from a patch has consequences on food intake (Fig. 4). Individuals that initiate movement spend approximately one-third less time (means ± SD: 31.81 ± 28.67%) on the patch than nonexcluded individuals. While at the periphery, these birds consume hardly any food [means ± SD: 0.06 ± 0.19 pecks per second (pps)], which represents a substantial drop in foraging relative to when they first entered the patch (means ± SD: 4.06 ± 0.71 pps). Food intake at the periphery is also lower relative to individuals that remain on the patch halfway through the time the group spends there (means ± SD: 4.22 ± 0.95 pps), relative to individuals that remain on the patch at the moment of the initiation (means ± SD: 3.5 ± 0.91 pps) and also relative to individuals that remain on the patch in the final 10 s before the last individual departs (means ± SD: 2.72 ± 0.49 pps). Thus, patches remain relatively rich, even when the last individual departs.

To determine whether the departures by the last individual on each patch are caused by depletion or not, we test whether these individuals express disproportionate movement characteristics that indicate that they are being pulled away from the patch. We use data from a group where all members were fitted with Global Positioning System (GPS) tags to calculate the distance from the last individuals at the moment of leaving the patch to the rest of the group and their speed while rejoining the group. These data show that the distance and speed as they leave the patch are both much greater than the individuals’ corresponding mean daily values (fig. S7), suggesting that the departing group is strongly pulling this last individual. These data do not support the hypothesis that dominant individuals that remain on the patch “lead from the back,” and rather show that these dominant individuals leave the patch to catch up with the rest of their group.

## DISCUSSION

It is well established that the distribution of resources can shape the social environment of group-living animals (*14*–*16*). Here, we extend this knowledge by showing that the distribution of resources can also determine who contributes to collective decision-making. In vulturine guineafowl, dominant males are typically more successful at initiating movement from dispersed resources. A dominance bias in leadership has also been observed in some mammals, including Japanese macaques (*Macaca fuscata*) (*17*) and wolves (*Canis lupus*) (*18*). Such outcomes can arise because larger individuals require more energy (*19*), and individuals that have a higher energetic demand, or are in a state of hunger, are more driven to move toward new resources. Such a mechanism has been found in groups in a range of species, including spotted hyenas (*Crocuta crocuta*) (*20*), plains zebras (*Equus burchellii*) (*21*), and schools of fish (*Rutilus rutilus*) (*22*). Given that male vulturine guineafowl are approximately 20% heavier than females, they are also presumably more motivated to find food, resulting in a greater tendency for them to initiate movements to new patches. However, when groups of vulturine guineafowl forage on monopolizable patches, dominant individuals exclude subordinates, causing the leadership to switch to subordinates. Being excluded from a patch reduces subordinates’ food intake (Fig. 4, A and B), which is likely to cause them to become more motivated to move to a new resource. Thus, our findings across both types of resources are consistent with the “leadership according to need” hypothesis (*7*), whereby motivation to forage shifts individuals’ priorities from group cohesion to finding new resources (*23*).

While our study demonstrates a switch in leadership under nonmonopolizable and monopolizable resources, we were limited to investigating decision-making dynamics in natural versus experimental patches. Nevertheless, our results raise interesting questions about the diversity of decision-making mechanisms that groups can express and how mechanisms might fluctuate in response to the types of resources group-living animals encounter. Future work would benefit from investigating how leadership emerges across a gradient of patch richness and distributions. One key future direction that warrants further exploration is whether there are critical transitions along the gradient of resource distributions or patch richness values at which leadership switches to being driven by subordinates.

Our data suggest that departures from monopolizable patches are not driven by food depletion but rather by social processes. In general, initiations take place when a large proportion of individuals have been displaced to the periphery (Fig. 4C), where they have no opportunity to forage (Fig. 4B). At this moment, the patch remains profitable, as birds that remain on it still peck more often than they do when the last bird leaves (Fig. 4B). Thus, departures are not driven by food because patches appear to remain profitable when the initiation takes place. Further, the last group members also appear to leave the patch while it remains productive, with these birds remaining on the patch until the last moment and then running to catch up with the group, confirming that they are being pulled away from the patch rather than moving on due to resource depletion. This finding raises interesting questions about how groups forage at a landscape scale. For example, optimal foraging theory suggests that larger groups of identical foragers should deplete patches more extensively than smaller groups, before leaving (*24*). However, groups of vulturine guineafowl, which live in a highly structured society, leave monopolizable patches, while the patch remains productive. A recent study on white-faced capuchins (*Cebus capucinus*) also showed that groups depleted patches differently, closer to the center versus closer to the periphery of their home ranges (*25*). Together, these results contribute to an emerging literature highlighting that within- and between-group social processes might alter the predictions of optimal foraging models.

In examining the social process during group departures, we found that subordinate vulturine guineafowl depart from the periphery of the patch once a critical number of birds accumulates there and that this critical number is not related to group size. This behavior is similar to what has been described as “quorum” decision-making, whereby the probability of group members performing a behavior (e.g. making a choice) is an increasing function of the number of group members that already performed it (*26*, *27*). Quorum decision-making has previously been shown to allow groups to be more accurate when choosing among options, such as between richer patches (*28*) or safer shelters (*29*). Our data therefore extend this previous work by also suggesting that a quorum might provide groups with a mechanism by which they can escape an option when it does not sufficiently benefit the average group member.

Dominant individuals following subordinates could be due to producer-scrounger dynamics (*30*, *31*), where subordinate individuals discover new resources that are then monopolized by dominants. An example of dominance-based producer-scrounger dynamics comes from flocks of Arctic barnacle geese (*Branta leucopsis*). Subordinate geese explore the environment for rich patches and are then excluded from these by dominant individuals that continue to monopolize the patch over the subsequent year (*32*). The behavior of dominant barnacle geese contrasts with those of dominant vulturine guineafowl. We found that the departure by subordinate vulturine guineafowl prompts dominant group members to abandon the patch, moving fast to rejoin the group while it remains “on the move” (movie S1). Thus, what pulls dominant vulturine guineafowl is the need to maintain group cohesion, as the distance to the group becomes large and not the opportunities to scrounge on new resources discovered by subordinates.

We show how shared decision-making can allow the average group member to reduce the costs that arise from the monopolization of resources by dominants (*33*). Previous research on chacma baboons (*Papio ursinus*) suggested that dominant individuals were leaders because groups moved to monopolizable food resources (*1*), while a later study on olive baboons (*Papio anubis*) suggested that, in general, baboon decisions were shared (*3*). Our results unite the findings from these two studies by revealing that who is most influential can be determined by prior context rather than by where groups are moving to next. In this way, shared decision-making allows groups to balance the needs of their members. When dominant individuals exert too much control, their actions promote greater influence by deprived group members in the subsequent decisions that determine what the group does next.

## MATERIALS AND METHODS

### Experimental design

The research took place at the southern part of the Mpala Research Center (0°17′N, 37°52′E), a conservancy area of nearly 20,000 ha of savannah and dry woodland habitats in Laikipia district, central Kenya. Within the study area, there are 18 color-banded and GPS-tracked groups of vulturine guineafowl (*A. vulturinum*), which we have been studying since August 2016. By GPS tagging and color banding several individuals in each group and monitoring membership composition twice a week, we have found that vulturine guineafowl live in a multilevel society that consists of stable groups that associate preferentially with specific other groups, and each contain many breeding males and females (*9*). Some of the subadults stay in their natal group, and others disperse. Breeding vulturine guineafowl split from their groups in the wet seasons; we collected our data during dry seasons when groups stay together and move cohesively. Our study area has two dry seasons (from mid-December to the end of March and from June to September) and two wet seasons (from April to May and from October to December) (*34*).

Three of our 18 study groups, containing 22 [habituated group 1 (HG1)], 24 (HG2), and 15 (HG3) adult individuals, have been habituated to human presence, and all individuals are marked with unique color bands. We collected data from HG1 during February and March 2017, data from HG2 from July to September 2018, and data from HG3 from January to March 2019. During these study periods, we also collected data from four additional, nonhabituated groups using camera-tracking techniques. In table S1, we summarize the data collection techniques we used to study each of these seven groups.

Vulturine guineafowl chicks join the social group of their parents after they hatch and then follow the adults. In all our analyses, we include only adult and subadult individuals, as our field observations suggest that chicks (<10 months old) always follow the adults and do not initiate movements. In the season when we collected the data for HG1, there were no chicks in the group, and all subadults had hatched at least 13 months before the study season. When we collected data from HG2, the group had 17 chicks that were approximately 3 months old. When we studied HG3, they had six chicks that were approximately 7 months old.

### Data collection

#### Dominance hierarchy

To estimate the dominance hierarchy in HG1, HG2, and HG3, we recorded the winners and losers (*35*, *36*) of the agonistic interactions observed while following the groups on foot. We recorded interactions using an all-occurrence sampling method (*37*). We defined a range of dyadic interactions as agonistic or dominance-related (table S3). We used these winner-loser interactions to calculate and rank each individual by Elo scores (*38*). Because our long-term data from HG1 suggest that dominance ranks are maintained across years, we assumed dominance ranks to be stable over the short period of the study (2 months for HG1 and 3 months for HG2). This allowed us to use a routine (*39*) to randomize the ordering of interactions and generate a more robust estimate of dominance rank. We created 1000 replicated datasets and calculated the mean and 95% confidence intervals of ranks of individuals based on Elo score, using the R Package “aniDom” (*40*).

#### Movement initiation from nonmonopolizable food resources

We followed the HG1 from 8 February 2017 to 28 March 2017 for ca. 4.5 hours per day (totaling >200 observation hours). We recorded the identity of initiators from departures after the group was stationary for more than 3 min. These stationary periods typically involved feeding on seeds or grass roots that they naturally find on the ground. We defined initiators as individuals that moved more than 20 m away from the center of the group. We also recorded the departure order of each individual. The initiation was considered successful if all group members followed the direction of the initiator and nonsuccessful otherwise (if not all individuals followed or if the initiator and followers returned to the group). The HG1 is the only group that we could follow on foot for many hours as the group moves within the Mpala Research Center compound, an area where it is safe to walk due to electric fences that exclude predators and large mammals.

#### Interactions within and movement initiations from monopolizable patches

We created monopolizable patches to study how social interactions determined who initiated movement away from such resources. For every study day, we added a small quantity of seeds (9 g of seeds per individual) on the ground at dawn within a previously marked circle of 1.5-m diameter. Each focal group typically arrived at these known patches between 6:30 and 7:30 a.m. After a vulturine guineafowl group discovers a patch and visits it for 2 to 4 days, they keep visiting it every day as their first activity in the morning. Most days, groups came directly from their roost, although the patches were always located more than 150 m away from each of the groups’ roosting site. Upon arrival, all group members accessed the patch on every occasion and always very synchronously. While the group was feeding, we recorded (i) agonistic interactions within the patch, (ii) the time that each individual left the patch, (iii) the departure order and timing from the periphery of the patch, and (iv) any attempts of the excluded individuals to reenter the patch. Departure orders were defined as the sequence of individuals that passed the 20-m radius centered on the patch, following a successful movement initiation. Successful initiations required all individuals to follow the first departing bird (the initiator) toward a new destination. Across all 41 sessions on patches, we recorded only one unsuccessful initiation, which was attempted by the first excluded individual, while all others remained on the patch.

We recorded all of the data from HG1 on a voice recorder. For all other groups, we used cameras to extract data on the exact timing of the departure process (table S1).

#### Peck rates on monopolizable patches and at their periphery

To estimate food intake within and on the periphery of the patches, we analyzed videos using the “Elmedia player” application. For each of the five first videos from HG2 and HG3 (10 videos in total), we randomly selected a highly visible adult individual at the start of the video, another in the middle of the video, and one at the moment of the initiation (the middle was calculated based on the time the birds arrived on the patch and the time when the last individual left it). We also picked one visible individual among the last individuals to leave the patch, 10 s before the last individual left the patch. In each case, we counted the number of pecks within 5 s, and we only considered birds that were not aggressing someone and were not aggressed 10 s before and after counting the pecks. Then, we calculated the pecking rate as the average pecks per second at the start, when the birds first arrive on the patch, in the middle, and at the end.

We also randomly selected 10 individuals (one from each video) from the periphery of the patch that had been displaced by dominants and thus excluded from the resource, and we calculated their peck rate, as above. The focal individuals for these measures were not running away from the dominants but had remained at the periphery for at least 30 s, to avoid counting less pecks as an effect of a preceding dominance interaction. The focal individuals were also not engaged in directed movement.

#### GPS-tagged group

We caught and GPS-tagged all the members of the HG3 to show that groups of vulturine guineafowl move cohesively during the dry season and to calculate the distance and the speed of the last individuals leaving the patch from the rest of the group’s centroid. Details on how we caught and tagged this group are presented in (*9*). Trapping and tagging were performed under permission from the Kenya Wildlife Service (KWS/904) and from the Ethikrat Committee of the Max Planck Society. GPS tagging also allowed us to see where the group moved a few hours after leaving the patch. The GPS tags were programmed to work synchronously every 4th day, to allow their solar-powered battery to charge. We collected a total of 13 days’ data from 9 February 2019 to 29 March 2019.

### Data analysis

#### Movement initiation from nonmonopolizable food resources

We tested whether dominance rank could predict whether an individual would be a successful initiator in a given departure by running a GLM on departures of HG1 from nonmonopolizable resources. The response variable was binary (1: successful; 0: unsuccessful) for a given initiator, and the independent variable was the dominance rank.

#### The relationship between interactions on the monopolizable patch and departure orders

We tested whether dominance plays a mechanistic role in movement initiations in a social system where group decisions are shared. We used our data on agonistic interactions to test whether these interactions predicted the departure order from a patch. To do this, we fit an ordinal logistic regression model to our ordered response, which is the departure orders for each of the group members, using the R Package MASS (*41*). Our predictor variable was a binary variable, where 1 corresponded to cases where an individual was a loser of an agonistic interaction on the patch before the point of the initiation and 0 corresponded to cases that were not.

#### Camera-tracking experiments to investigate the timing of the departure process from monopolizable patches

We studied the timing of the departure process from the patches and estimated food loss and food intake for dominants and excluded individuals by video-tracking HG2, HG3, and four additional, nonhabituated groups of vulturine guineafowl. We collected these tracking data during February 2018, from July to September 2018, and from February to March 2019. We used one close-up camera to clearly record agonistic interactions on the patch and one wide-view camera to track the movement of the birds on the periphery of the patch and their departure from the periphery. The wide-view camera was placed 15 to 20 m away from the center of the patch. The exact positioning of the camera depended on the fine-scale habitat configuration. Using the Tracker application (*42*), we assigned a visual identification code to each moving bird, by watching, in parallel, the two videos collected from each session. In each video, we tracked all individuals to record the losers of agonistic interactions within the patch, the time spent on the patch, the time spent in the periphery of the patch (within a 15-m diameter centered on the patch), the departure time, and the departure order of each bird.

For HG2 and HG3, we managed to extract all these data from the videos. However, from the nonhabituated groups, we could only collect data on whether the initiator was aggressed and thus displaced from the patch before initiating and on the number of individuals in the periphery of the patch before initiating departure (see questions 11 and 12 from table S1). All members of the habituated groups were color-banded, and the observer (D.P.) could be close to the groups and could describe the departure process and all the interactions that took place. By contrast, the color bands were not always visible in the videos for all the members of the nonhabituated groups, and some group members were unbanded, meaning that we lost track of some individuals during the course of a trail. However, we could easily tell (i) whether the initiator was excluded from the patch and (ii) how many individuals were in the periphery of the patch when the first individual initiated movement.

#### Movements of the GPS-tagged group

We used high-resolution GPS data (1-Hz sampling continuously, every 4th day) from HG3 to estimate the pairwise distances between all group members, when the group was cohesive. On the basis of the distribution of the pairwise distances within the group (fig. S1), we classified the group as cohesive for all 5-min intervals when no pairwise distances exceeded 100 m. When the group was cohesive, we also calculated the group’s centroid every 5 min to estimate group position. We extracted the directional vector of the initiator from the patch by watching the animations of the GPS data using Google Earth Pro and the method described above to determine initiations. We also calculated the distance of the last individuals that left the patch from the rest of the group’s centroid (fig. S7). Last, we plotted the vector of the initiator and the tracks of the group’s centroid from early morning (6:00 a.m.) when the birds were leaving their roost until 2:00 p.m., as presented in fig. S4.

## Supplementary Material

http://advances.sciencemag.org/cgi/content/full/6/48/eaba5881/DC1

Movie S1

Adobe PDF - aba5881_SM.pdf

Shared decision-making allows subordinates to lead when dominants monopolize resources

## SUPPLEMENTARY MATERIALS

Supplementary material for this article is available at http://advances.sciencemag.org/cgi/content/full/6/48/eaba5881/DC1
