# Supplementary material for: Shared decision-making allows subordinates to lead when dominants monopolize resources
Source: Sci Adv. 2020 Nov 25;6(48):eaba5881. doi: 10.1126/sciadv.aba5881 (PMC7688327; doi:10.1126/sciadv.aba5881)
Supplement: http://advances.sciencemag.org/cgi/content/full/6/48/eaba5881/DC1 [file supp_6_48_eaba5881__index.html]

Science Advances | Science AdvancesAAASSearchScience AdvancesMenu

## Supplementary Materials

# Shared decision-making allows subordinates to lead when dominants monopolize resources

Danai Papageorgiou and Damien R. Farine

Download Supplement

**This PDF file includes:**

- Supplementary text
- Figs. S1 to S7
- Tables S1 to S7
- Legend for movie S1

**Other Supplementary Material for this manuscript includes the following:**

- Movie S1

**Files in this Data Supplement:**

- Adobe PDF - aba5881\_SM.pdf
- aba5881\_Movie\_S1.mp4
