# Supplementary material for: Shared decision-making allows subordinates to lead when dominants monopolize resources
Source: Sci Adv. 2020 Nov 25;6(48):eaba5881. doi: 10.1126/sciadv.aba5881 (PMC7688327; doi:10.1126/sciadv.aba5881)
Supplement: http://advances.sciencemag.org/cgi/content/full/6/48/eaba5881/DC1 [file supp_6_48_eaba5881__1.pdf]

Science Advances
AAAS

advances.sciencemag.org/cgi/content/full/6/48/eaba5881/DC1

Supplementary Materials for

Shared decision-making allows subordinates to lead when dominants monopolize resources

Danai Papageorgiou* and Damien R. Farine*

*Corresponding author. Email: dpapageorgiou@ab.mpg.de (D.P.); dfarine@ab.mpg.de (D.R.F.)

Published 25 November 2020, *Sci. Adv.* **6**, eaba5881 (2020)
DOI: 10.1126/sciadv.aba5881

The PDF file includes:

Supplementary text
Figs. S1 to S7
Tables S1 to S7
Legend for movie S1

Other Supplementary Material for this manuscript includes the following:

(available at advances.sciencemag.org/cgi/content/full/6/48/eaba5881/DC1)

Movie S1

Supplementary Text

Dominance hierarchy

We found a clear dominance hierarchy within vulturine guineafowl groups (Fig. 1, Fig. S2). The hierarchy of the habituated groups was very steep; the probability of the higher-ranked individual winning an agonistic interaction was 0.9 when the rank difference was only 1.

Movement initiations from non-monopolisable resources

All group members could initiate movement, but adult males higher in the dominance hierarchy were slightly more likely to be followed (Table S2). The reason why dominant individuals appeared to be more often among the initiators could be that males are 20-30% larger in body size, and therefore likely to be more motivate to lead. The observed differences in initiating group movements are more strongly defined in the Male-Female dichotomy rather than along dominance lines, because even individuals halfway down the male dominance hierarchy could initiate successfully. In addition, one female initiated frequently, and this female was the oldest member of the group. While we have no historical data on this individual, her leadership could reflect differences in her knowledge of the environment. Thus, while we found that there is stratification in leadership in this group, the differences do not suggest deviation away from shared decision-making, since all adult individuals were able to initiate movement successfully.

Time spent on the monopolisable patches

The mean duration of departure process from the periphery of the patch, from the onset of the initiation to the last individual was 1.23 minutes (SD= ±1.37, min= 0.85min, max= 4.78min) and the group members stayed on the patch for 8.16 minutes (sd=2.85).

The relation between dominance hierarchy and initiations from monopolisable patches

We found no consistency in the identity of the initiator of the movement away from the patch across initiation attempts (Table S4). That is, neither the dominant individual, nor other highly-ranked individuals, were responsible for most of movement initiations. This clearly differs from the expectations of despotic leadership.

The relation between agonistic interactions on monopolisable patches and departure orders

The ordinal logistic regression model revealed that the probability for an individual to occupy the first orders in a departure increases significantly if this individual had been the loser of an agonistic interaction on the patch (Fig. 3, Fig. S6, Table S5). The departure is not a direct effect of being the loser of an agonistic interaction, as shown by the distribution of the time lags between losing in an interaction (i.e. being displaced) and subsequently departing (Fig. 4a).

Supplementary Figures

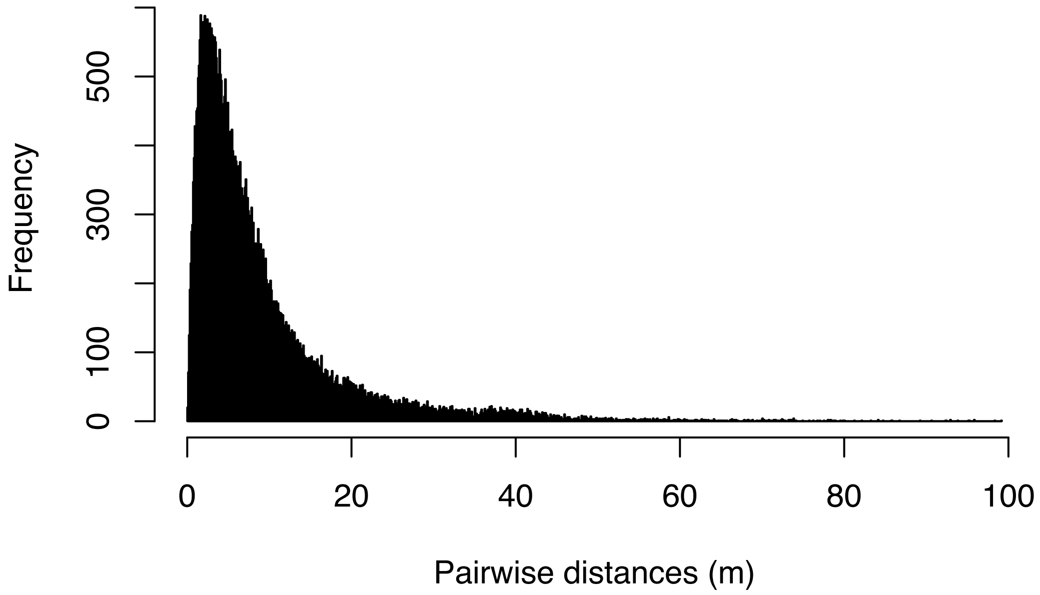

Fig. S1: Vulturine guineafowl groups move very cohesively in the non-breeding season. The distribution of the pairwise distances between all group members of HG3, which were all synchronously GPS tracked. For more information about vulturine guineafowl group cohesion see (*9*).

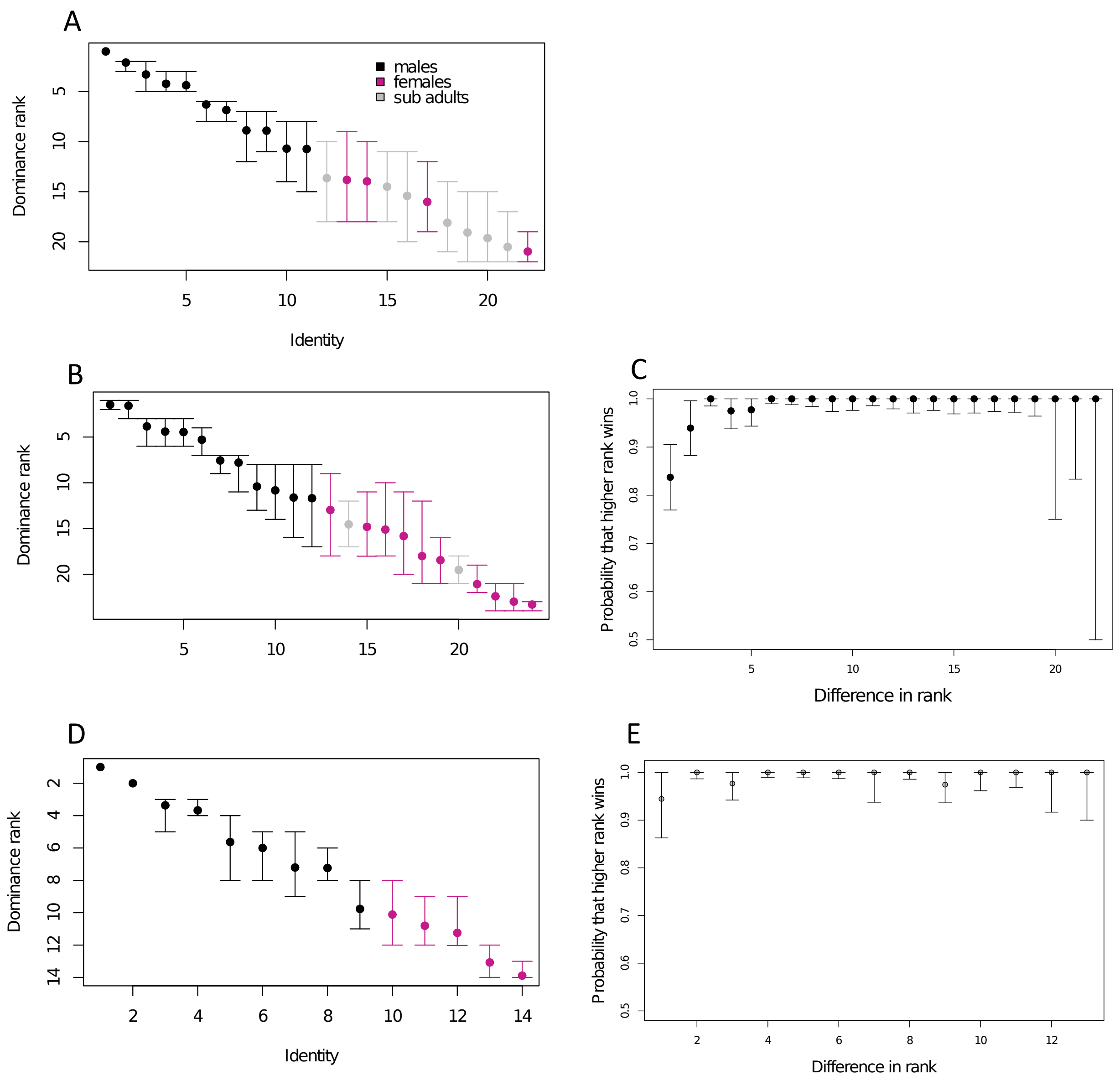

Fig. S2: Adult male vulturine guineafowl occupying the top of the steep dominance hierarchy within their groups reveals a steep within-group dominance hierarchy. Whiskers represent 95% confidence of rank estimates based on 1000 randomizations (*36*). **(A,B,D)** The dominance hierarchy of each of the three habituated groups (**A** for HG1, **B** for HG2 and **D** for HG3); **(C,E)** The probability of an individual to win an agonistic interaction, according to the difference in dominance rank for HG2 and HG3 respectively.

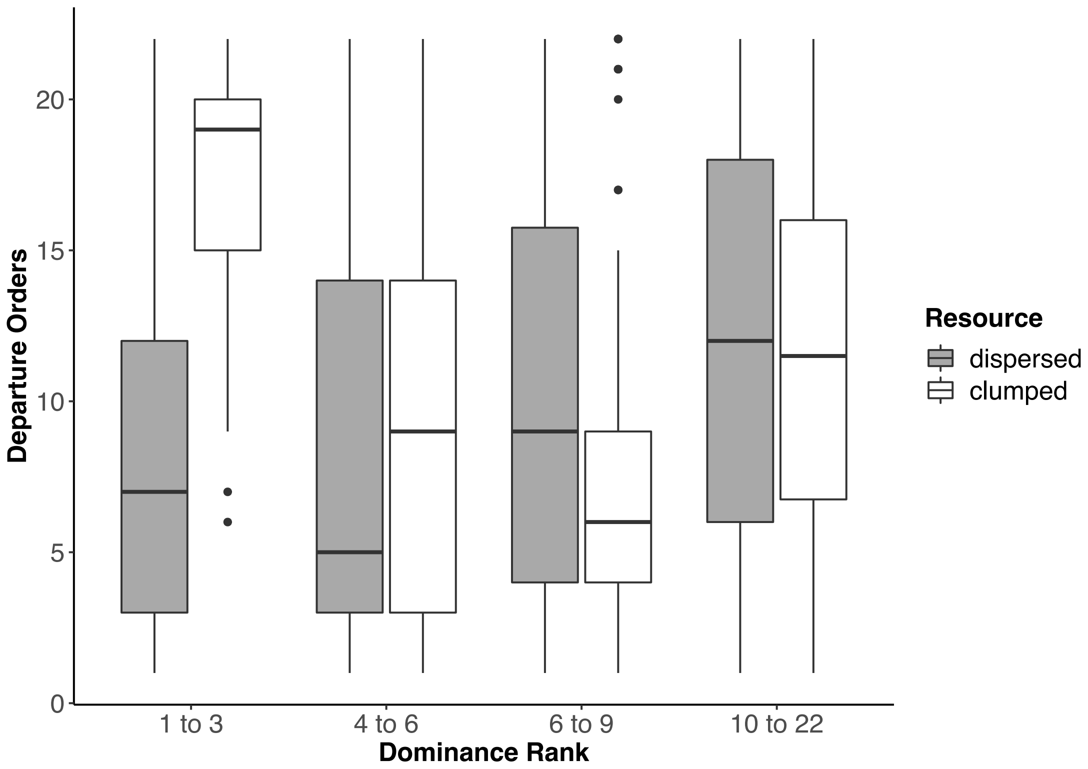

Fig. S3: Effect of resource type on who initiates. The orders that individuals occupy in collective departures of the HG1, according to their dominance rank when departing from non-monopolisable resources (dispersed) and when departing from monopolisable patches (clumped). The category 1 to 3 are the most dominant group members, and "1" in the y-axis refers to first departing individual.

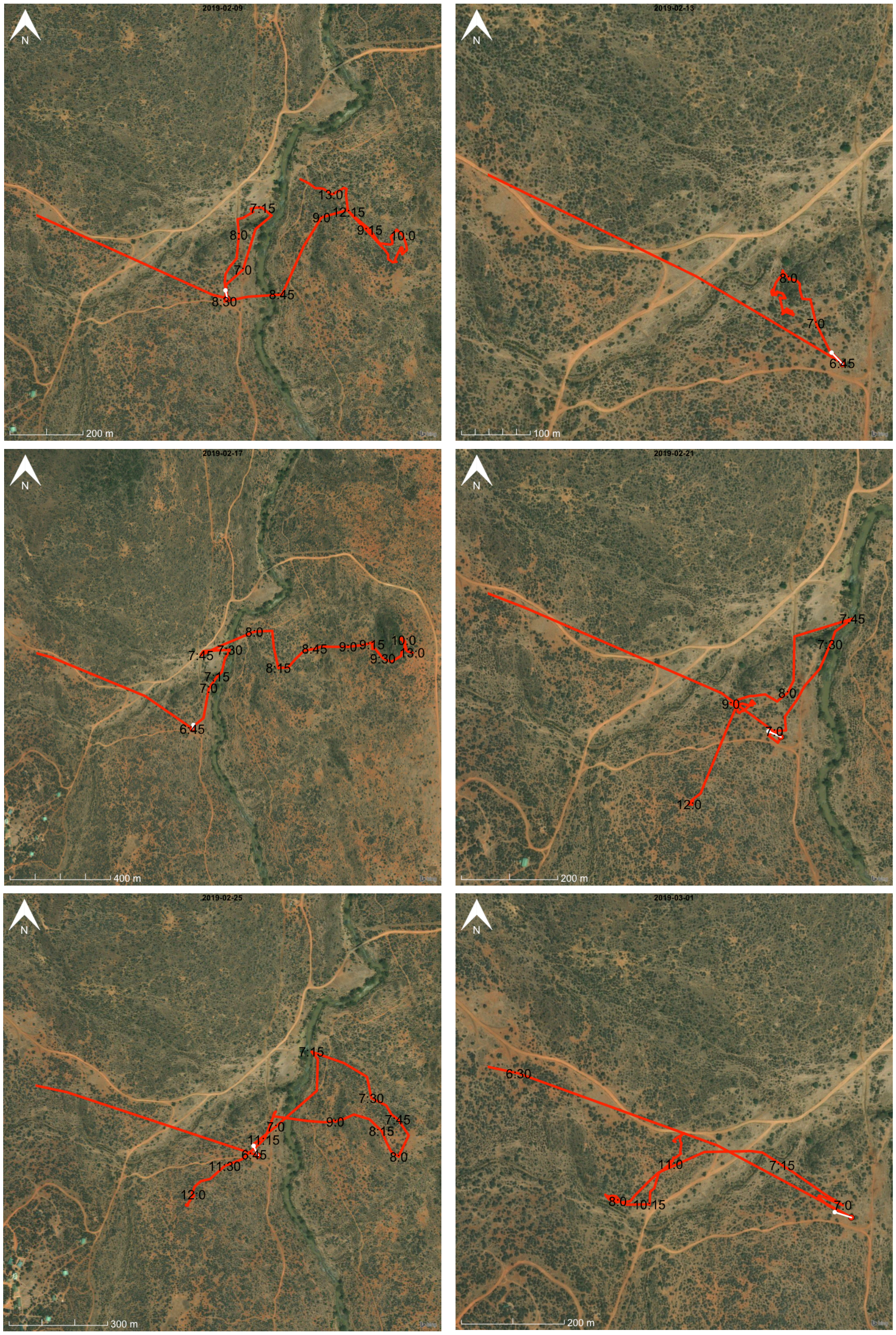
2019-02-09
N
7:15
8:0
7:0
8:30
8:45
13:0
9:0
12:15
9:15
10:0
200 m
2019-02-13
N
8:0
7:0
6:45
100 m
2019-02-17
N
8:0
7:45
7:30
7:15
7:0
6:45
8:15
8:45
9:0
9:15
9:30
10:0
13:0
400 m
2019-02-21
N
7:45
7:30
8:0
9:0
7:0
12:0
200 m
2019-02-25
N
7:15
7:30
7:0
9:0
7:45
8:15
11:15
6:45
8:0
11:30
12:0
300 m
2019-03-01
N
6:30
11:0
7:15
8:0
10:15
7:0
200 m

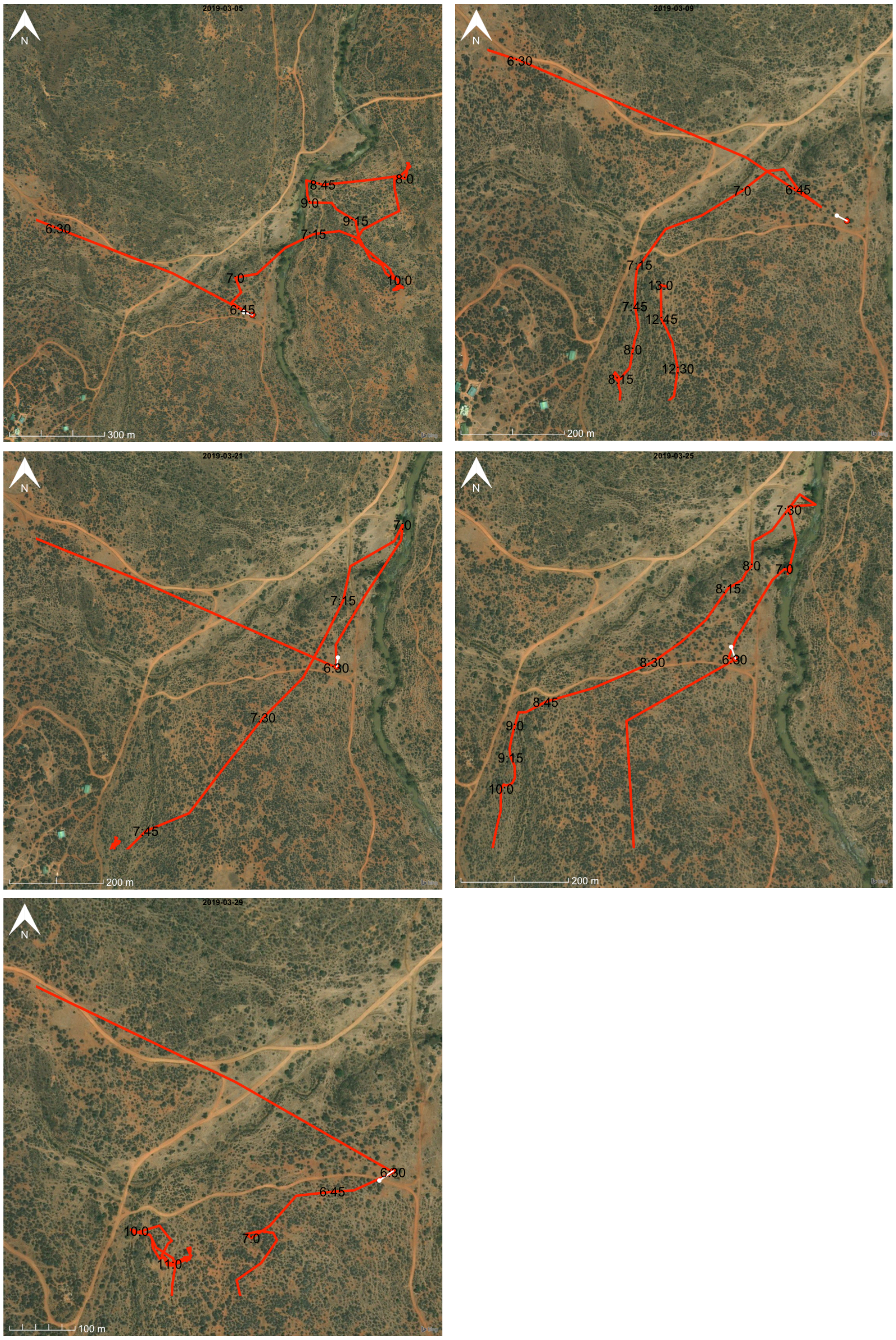

Fig. S4: For most of the days, the direction taken by the initiator determined where the group spent the rest of the morning. The daily tracks of the calculated centroid of the GPS tagged HG3 for every five minutes, before visiting the patch (between 6:20AM and 07:05AM) in the morning and until 14:00. The red dot represents the patch and the white line ending in a white dot represents the direction of the initiator. Microsoft Bing satellite maps reprinted with permission from Microsoft Corporation.

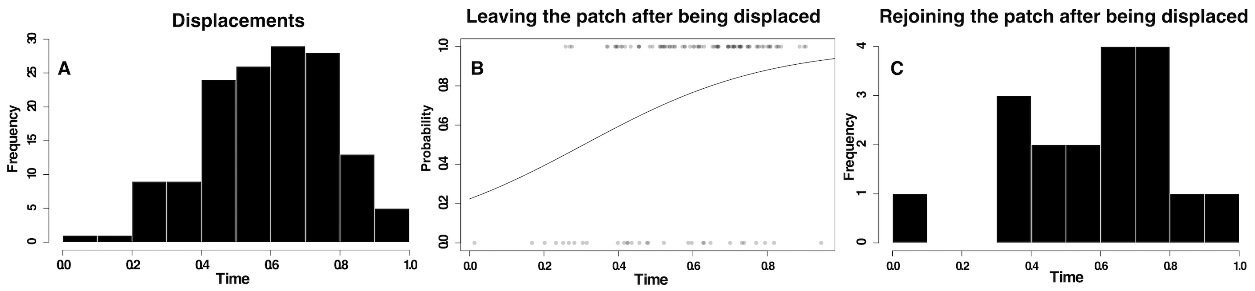

Fig. S5: The timing of displacements, leaving and rejoining monopolisable patches. (A) The distribution of the occurrence of agonistic interactions (i.e. displacements) from the beginning (0) until the end (1.0) of a session. **(B)** The probability of leaving the patch right after having being displaced as a function of the timing of a session. **(C)** The distribution of the events of rejoining the patch after having being displaced as a function of the timing of a session.

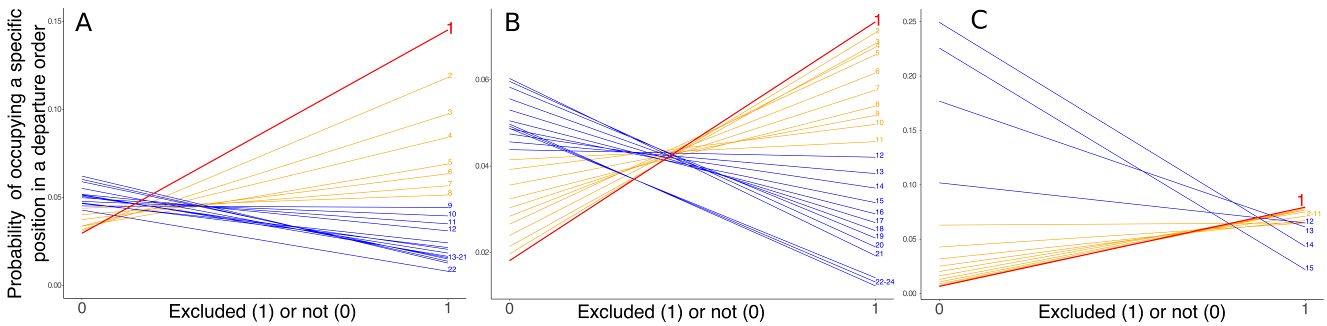

Fig. S6: Individuals that depart first from monopolisable patches are those who were excluded (i.e. displaced) by dominants in (A) HG1, (B) HG2, (C) and HG3. The predicted probability for an individual to occupy a particular order in a departure from the patch, according to whether this individual had been excluded from the patch (red and orange) or not (blue), as shown by an ordinal logistic regression model.

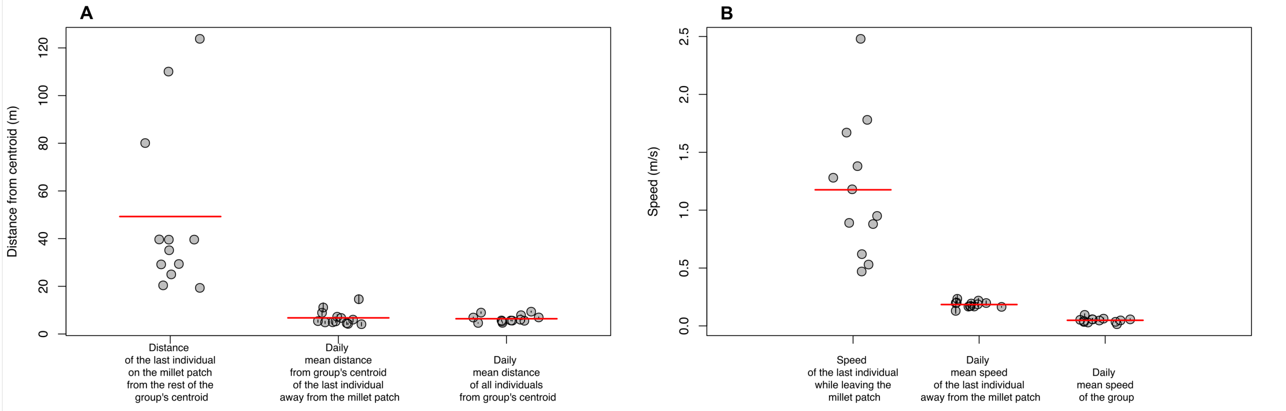

Fig. S7: The last individual that departs from the monopolisable patch is further away from the group's centroid and moves faster to catch up with the group, when compared to normal group movements. (A) The distance of the last individual to leave the patch from the rest of the group's centroid at the moment when it starts moving towards the rest of the group, the daily average distance of this individual from the rest of the group's centroid and the average distance of the group's centroid for each group member of HG3, calculated using the high resolution and synchronous GPS tracking for each of the 13 days that the group was tracked. **(B)** As in (A) but for speed.

Supplementary tables

	Question	HG1[1,4]	HG2[2,4]	HG3[2,3,4]	G4[2]	G5[2]	G6[2]	G7[2]	Figure	Table	Sample size
**1**	Initiations from scattered food and resting resources[1,4]	x							1b<br>S3	S2	183 departures
**2**	Dominance hierarchy[1,2,4]	x	x	x					1a<br>S2	S3	2213 interactions
**3**	Consistency in the identity of the initiator from the patch[2,4]	x	x	x						S4	35 departures
**4**	Dominance rank and the propensity to initiate from the patch[2,4]	x	x	x						S5	
**5**	Exclusion from the patch and probability to occupy a specific order in a departure[1,2,4]	x	x	x					3<br>S5	S6	29 departures
**6**	Timing of the first displacement from the patch [2,4]		x	x							10 departures
**7**	Time spent on the patch and duration of the departure process[2,4]		x	x							
**8**	Pecking rates[2,4]		x	x					4b		
**9**	Time lags between agonistic interactions and departures from the patch [2,4]		x	x					4		
**10**	Timing of dsplacements and re-joining[2]								S4		
**11**	Initiator excluded or not from the patch [1,2]	x	x	x	x	x	x	x			41 departures
**12**	Number of individuals in the periphery of the patch before initiating[2]		x	x	x	x	x	x			22 departures
**13**	Group cohesion[3]			x					S1		13 days
**14**	Where the group goes after departing from the patch [3]			x					S7		13 departures
**15**	Distance and speed of the last individuals leaving the patch from the rest of the group's centroid[3]			x					S6		

[1] Following on foot and voice recording
[2] Camera tracking and voice recording
[3] GPS tracking
[4] All members were colour banded

Table S1. Summary of the data and groups available for addressing each question. We combined data from three habituated and from 4 non-habituated groups to answer 14 questions, but not all questions could be addressed with the data from all groups.

	Estimate	Standard Error	z value	p value
(Intercept)	1.41	0.3	4.77	**<0.001**
rank	-0.06	0.22	-2.83	**<0.001**

Table S2. Higher-ranked individuals are more successful in initiating group movements from non-monopolisable food resources. The results of the binomial GLM testing the effect of dominance rank on the probability of being followed by the rest of the group when departing from non-monopolisable food resources. Data include 183 group departures from HG1.

	Code	Description
1	CHA	Ind A chases ind B
2	PEC	Ind A pecks ind B on the head or on body
3	TAI	Ind A grabs B by tail or body feathers
4	SPI	Ind A grabs B by the tail feathers and spins them around
5	GAP	Ind A gapes at ind B (similar to chase display but without chasing). Ind B recedes
6	DIS	Ind A displaces ind B from foraging or resting (e.g. sand-bathing) spot
7	FIG	Ind A attacks and B fights back, flying up in the air, flapping wings and kicking
8	SUV	Ind A presents submissive falling in front of individual B. Usually observed in chicks, but also between adults when on the move and accompanied by a "crying call" and sometimes repeated in zig-zag. (eliciting little response from B)
9	SUB	Ind A submissive caress around the chest of individual B (often triggered by an agonistic behaviour by B)

Table S3. Overview of agonistic interactions. We identified 9 different types of agonistic interactions that guineafowl presented. In all but "8" and "9", individual A was regarded as the winner of the interaction and B as the loser.

HG1 Individual	HG1 Dominance Rank	HG1 Successful Initiations	HG2 Individual	HG2 Dominance Rank	HG2 Successful Initiations	HG3 Individual	HG3 Dominance Rank	HG3 Successful Initiations
KKRK	1	0	WT015	1	0	BLAK	1	0
WKOW	2	0	WT022	2	0	BABA	2	0
OOBB	3	0	WT016	3	0	LARY	3	0
OOOY	4	0	WYYG	4	0	GOAL	4	1
WBKR	5	5	YYWK	5	0	YGGK	5	0
YOKO	6	0	WT020	6	0	ALLY	6	0
BROB	7	0	YRGR	7	0	BYYW	7	0
KOBR	8	1	WT011	8	0	LOOK	8	0
RROR	9	1	WKOO	9	0	RWGO	9	0
RRYY	10	1	WT012	10	0	LOGO	10	0
WKOR	11	0	WT021	11	1	BAGG	11	1
BYWW	12	0	WT014	12	1	WAYK	12	0
GRWY	13	0	WT018	13	1	YAYK	13	0
YOBK	14	1	RGOB	14	0	RGOB	14	0
BYYO	15	1	WT013	15	1	OBYR	15	0
OWRB	16	0	WPPW	16	1	RALY	16	0
BYOO	17	0	WPOK	17	1	PWPO	17	2
ROOK	18	2	WT005	18	1	RGWO	18	1
ROWO	19	0	PBYK	19	0			
GKGK	20	0	PORK	20	0			
WBBB	21	5	OBYR	21	1			
KKBY	22	2	YYPG	22	0			
			GOYW	23	1			
			PWPO	24	0			

Table S4. Summary of the observation data. The number of times each individual from the three habituated groups of the study initiated movement away from the patch and their dominance rank. For two of the 11 collective departures we recorded from Group 2 we could not identify the initiator from the video.

(a)	Value	Std. Error	t value	p value
Intercept	-1.71	0.18	-9.57	**<0.001**
1|2	-3.63	0.22	-16.51	**<0.001**
2|3	-2.88	0.18	-15.87	**<0.001**
3|4	-2.42	0.16	-14.71	**<0.001**
4|5	-2.07	0.15	-13.42	**<0.001**
5|6	-1.78	0.15	-12.16	**<0.001**
6|7	-1.52	0.14	-10.83	**<0.001**
7|8	-1.27	0.13	-9.42	**<0.001**
8|9	-1.03	0.13	-7.92	**<0.001**
9|10	-0.80	0.13	-6.36	**<0.001**
10|11	-0.57	0.12	-4.69	**<0.001**
11|12	-0.34	0.12	-2.87	**<0.001**
12|13	-0.11	0.12	-0.90	0.37
13|14	0.12	0.12	1.01	0.31
14|15	0.35	0.12	2.98	**<0.001**
15|16	0.60	0.12	4.96	**<0.001**
16|17	0.82	0.13	6.53	**<0.001**
17|18	1.05	0.13	8.02	**<0.001**
18|19	1.34	0.14	9.47	**<0.001**
19|20	1.71	0.16	10.74	**<0.001**
20|21	2.23	0.19	11.49	**<0.001**
21|22	3.19	0.30	10.79	**<0.001**

**(b)**	**Value**	**Std. Error**	**t value**	**p value**
Intercept	-1.72	0.20	-8.45	**<0.001**
1|2	-3.49	0.24	-14.83	**<0.001**
2|3	-2.75	0.19	-14.27	**<0.001**
3|4	-2.29	0.17	-13.25	**<0.001**
4|5	-1.94	0.16	-12.12	**<0.001**
5|6	-1.66	0.15	-11.00	**<0.001**
6|7	-1.41	0.14	-9.80	**<0.001**
7|8	-1.17	0.14	-8.52	**<0.001**
8|9	-0.94	0.13	-7.13	**<0.001**
9|10	-0.73	0.13	-5.71	**<0.001**
10|11	-0.52	0.12	-4.18	**<0.001**
11|12	-0.31	0.12	-2.56	**0.01**
12|13	-0.10	0.12	-0.84	0.40
13|14	0.08	0.12	0.71	0.48
14|15	0.27	0.12	2.28	0.02
15|16	0.48	0.12	3.96	**<0.001**
16|17	0.71	0.13	5.61	**<0.001**
17|18	0.95	0.13	7.20	**<0.001**
18|19	1.24	0.14	8.75	**<0.001**
19|20	1.62	0.16	10.14	**<0.001**
20|21	2.15	0.19	11.02	**<0.001**
21|22	3.11	0.30	10.50	**<0.001**

**(c)**	**Value**	**Std. Error**	**t value**	**p value**
Intercept	-1.46	0.35	-4.22	**<0.001**
1|2	-3.99	0.51	-7.78	**<0.001**
2|3	-3.24	0.40	-8.06	**<0.001**
3|4	-2.77	0.36	-7.79	**<0.001**
4|5	-2.40	0.33	-7.35	**<0.001**
5|6	-2.10	0.31	-6.85	**<0.001**
6|7	-1.83	0.29	-6.31	**<0.001**
7|8	-1.60	0.28	-5.73	**<0.001**
8|9	-1.38	0.27	-5.13	**<0.001**
9|10	-1.17	0.26	-4.51	**<0.001**
10|11	-0.97	0.25	-3.84	**<0.001**
11|12	-0.77	0.24	-3.14	**<0.001**
12|13	-0.57	0.24	-2.39	**0.02**
13|14	-0.38	0.24	-1.61	0.11
14|15	-0.19	0.23	-0.80	0.43
15|16	0.01	0.23	0.04	0.97
16|17	0.21	0.24	0.90	0.37
17|18	0.43	0.24	1.79	0.07
18|19	0.67	0.25	2.71	**0.01**
19|20	0.94	0.26	3.65	**<0.001**
20|21	1.26	0.28	4.55	**<0.001**
21|22	1.66	0.31	5.32	**<0.001**
22|23	2.07	0.36	5.76	**<0.001**
23|24	2.71	0.47	5.81	**<0.001**
24|25	4.35	1.01	4.32	**<0.001**

(d)	Value	Std. Error	t value	p value
Intercept	-2.56	0.70	-3.68	**<0.001**
1|2	-5.01	0.81	-6.20	**<0.001**
2|3	-4.24	0.74	-5.70	**<0.001**
3|4	-3.74	0.72	-5.21	**<0.001**
4|5	-3.35	0.70	-4.76	**<0.001**
5|6	-3.01	0.69	-4.34	**<0.001**
6|7	-2.70	0.69	-3.93	**<0.001**
7|8	-2.40	0.68	-3.53	**<0.001**
8|9	-2.11	0.67	-3.13	**<0.001**
9|10	-1.81	0.67	-2.72	**0.01**
10|11	-1.50	0.66	-2.26	**0.02**
11|12	-1.12	0.65	-1.72	0.09
12|13	-0.63	0.63	-0.99	0.32
13|14	0.10	0.59	0.17	0.87
14|15	1.23	0.63	1.94	**0.05**

Table S5. Agonistic interactions predict initiation order away from monopolisable patches. (a) The summary of the ordinal logistic regression model for HG1 and HG2, which were of similar size. **(b)** The summary of the ordinal logistic regression model only for HG1. **(c)** The summary of the ordinal logistic regression model only for HG2. **(d)** The summary of the ordinal logistic regression model for HG3. The logical way of testing the hypothesis is to make the exclusion the predictor variable and the probability of occupying the response variable, as done here. But, because the data are difficult to interpret in this way, we present just the raw data in the main text in Fig. 3.

	Estimate	Standard Error	t value	p value
Intercept	1.65	0.10	16.62	**<0.001**
Initiator	-0.19	0.34	-0.58	0.57
Not Excluded	-1.15	0.15	-7.52	**<0.001**

Reference: Excluded

Table S6. Non-excluded individuals spent significantly less time at the periphery of the monopolisable patch than excluded individuals. The results of the GLM testing the time spent on the periphery by individuals that were either excluded from the patch (reference), or were not excluded or they initiated movement regardless if they were excluded or not. Data include 10 group departures from HG2 and HG3.

	Estimate	Standard Error	t value	p value
Intercept	4.06	0.22	18.17	**<0.001**
Middle	0.16	0.32	0.51	0.62
Initiation	-0.56	0.32	-1.77	0.08
End	-1.34	0.32	-4.24	**<0.001**
Periphery	-4	0.32	-12.66	**<0.001**

Reference: Start

Table S7. Individuals at the periphery of the monopolisable patch consumed significantly less food than individuals that remained on the patch. The results of the GLM testing the pecks per second for the birds that first accessed the patch (0) compared to birds that remained on the patch in the middle (0.5), at the end of the session (1) and in the periphery of the patch. Data come from HG2 and HG3.

Caption for Movie S1

The visualization of the GPS data for one day (25.03.2019) when HG3 visited the monopolisable patch following the process described in Fig.2 of the main text. The last individual that leaves the patch to catch up with the rest of the group (light green) is the alpha male.
